# *Henneguya* sp. in yellowfin goby *Acanthogobius flavimanus* from the San Francisco Estuary

**DOI:** 10.1186/2193-1801-2-420

**Published:** 2013-08-29

**Authors:** Dolores V Baxa, Alison Stover, Mark Clifford, Tomofumi Kurobe, Swee J Teh, Peter Moyle, Ronald P Hedrick

**Affiliations:** School of Veterinary Medicine, Department of Anatomy, Physiology, and Cell Biology, University of California, Davis, CA 95616 USA; Wildlife, Fish, and Conservation Biology, Center for Watershed Sciences, University of California, Davis, CA 95616 USA; California Department of Fish and Wildlife, #3 North Old Stage Road, Mt. Shasta, CA 96067 USA; School of Veterinary Medicine, Department of Medicine and Epidemiology, University of California, Davis, CA 95616 USA

**Keywords:** Yellowfin goby, Myxozoan, San Francisco Estuary, *Henneguya*

## Abstract

Myxozoan spores were observed in yellowfin goby *Acanthogobius flavimanus* collected from Suisun Marsh, San Francisco Estuary (SFE). Although histopathological changes associated with the parasite were not observed, the spores formed plasmodia that partially blocked the gastric and intestinal mucosa and gut lumen and may affect the perfomance and survival of the yellowfin goby. Morphological features of the spores resembled *Henneguya* sp. and molecular analysis of the 18S ribosomal DNA (Domain III) confirmed close similarity to *H. rhinogobii* and *H. pseudorhinogobii* isolated from the Japanese freshwater goby. The yellowfin goby myxozoan however, is likely an undescribed species based on phylogenetic analysis and morphologic features. Detailed description of vegetative and spore stages are currently lacking for proposal to a new species of *Henneguya*. A specific PCR test was developed, which confirmed a 100% prevalence of the parasite among randomly collected gobies in group 1 (N = 30) and group 2 (N = 15) at termination of the study at one month in captivity. The myxozoan was also detected from 18 gobies (12%) that died in the first group within two weeks in captivity. Apparently healthy gobies that served as controls did not reveal the presence of the myxozoan by PCR. This study documents the occurrence of a potentially new species of myxozoan in the yellowfin goby and underscores the detection of a parasitic infection in an introduced fish in the SFE. Although the pathogenesis of the myxozoan was not assessed and the prevalence as reported here is restricted to a comparatively small collection site in Suisun slough, the reemergence, identification, and ecological relevance of the parasite on goby populations in the SFE may be investigated in the future using the specific diagnostic tool developed in this study.

**Electronic supplementary material:**

The online version of this article (doi:10.1186/2193-1801-2-420) contains supplementary material, which is available to authorized users.

## Background

The San Francisco Estuary (hereafter SFE) is the largest estuary on the U.S. Pacific Coast. It provides drinking water to 25 million California residents, irrigation water to one of the most productive agricultural economies worldwide, and an open−water habitat to hundreds of native plants and aquatic organisms including 212 exotic and 123 cryptic species of unknown origin (Cohen and Carlton 1998Service RF 2007). Although the SFE is one of the most invaded (Cohen and Carlton 1998) and perturbed (Nichols et al. 1986) water bodies worldwide, determining the occurrence of pathogens and diseases on native and introduced fishes in this estuarine ecosystem has not been a goal of the various fish monitoring surveys of the Sacramento San Joaquin Delta. The yellowfin goby (hereafter goby) *Acanthogobius flavimanus* is native to northern Asia and Japan (Akihito et al. 2002) and has been an abundant bottom fish (Moyle 2002Feyrer and Healey 2003) following its introduction in the SFE via ballast water and first detection in 1963 (Brittan et al. 1963Dill and Cordone 1997).

This study describes the occurrence and identification of a myxozoan parasite among juvenile yellowfin gobies collected during a monitoring program that assessed the distribution and abundance of various fish species in Suisun Marsh, a critical rearing habitat for juvenile fishes in the SFE. Our goals are two-fold: 1) identify the myxozoan using a molecular-based approach and 2) evaluate the prevalence of infections among collected gobies by designing and using a PCR test specific to the myxozoan. The development and application of this molecular tool is an important first step for specific identification and assessment of the parasite among gobies and other fish species of economic and ecologic importance in the SFE that may be infected with the myxozoan.

## Results

**Figure 1:**
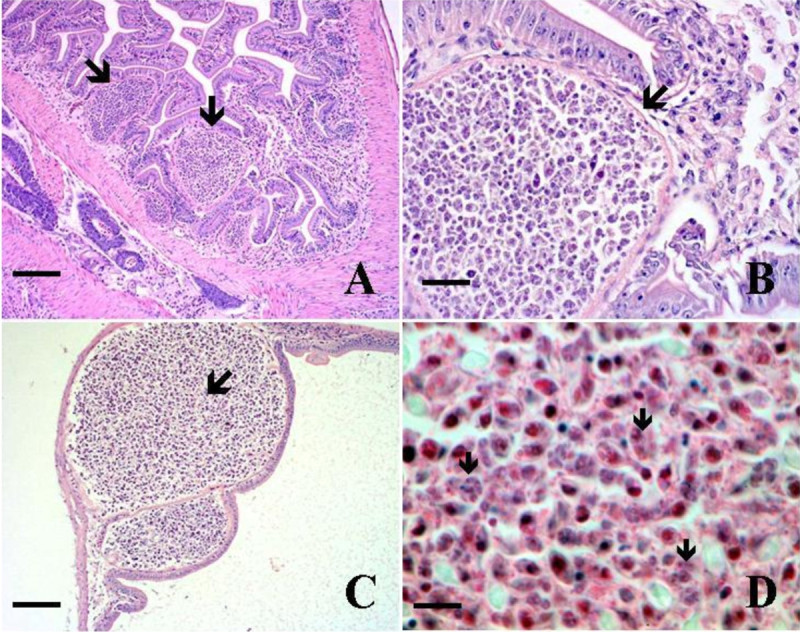
**Myxozoan spores in the yellowfin goby. A)** Spore plasmodia (arrows) partially blocking the intestinal cavity, Scale bar = 333 μm, **B)** Closer view of spores showing a thin line outside the plasmodium indicating the original intestinal lining (arrow), Scale bar = 67 μm, **C)** Spores (arrow) in the stomach, Scale bar = 333 μm, and **D)** Closer view of spores (arrows) in the stomach. Scale bar = 22 μm.

Eighteen of 151 gobies (average size = 1.5 gm, 50 mm) in the first group died within two weeks of captivity, whereas no mortalities occurred in the second group (N = 55, average size: 10.0 gm, 100 mm) until termination of the study after one month in captivity. Although none of the fish died in captivity from the second group, the 15 fish examined for histopathology showed variable presence of the parasite and all were positive by PCR including the 10 fish sampled from group 1. Spores were formed within plasmodia in the gastric and intestinal mucosa that extended into the gut lumen and partially blocked the intestinal cavities (Figure 1) however, microscopic changes associated with the parasite were not observed. Stained sections (hematoxylin and eosin) showed variable presence of myxozoan spores from stomach and intestines but difficult to confirm for vegetative stages. Moribund fish (N = 18) that succumbed to infection in the first group (12%) revealed the presence of spores in the intestine and stomach but gross clinical signs of infection were not observed. In addition, these moribund fish did not show the presence of the myxozoan in other tissues such as the liver, kidney, and gills. Examination for ectoparasites and culture of kidney and spleen tissues on general isolation medium (e.g. blood agar plate) did not reveal any bacterial growth as potential causes of mortality.

Initial validation of the PCR assay did not show any cross-reaction with *Myxobolus* sp. and *M. cerebralis* (Kent et al. 2001). Other myxozoans need to be tested in the future to further confirm the specificity of the PCR method. Using the specific PCR assay, the prevalence of infections with the myxozoan was assessed from randomly collected fish remaining in both groups at the end of the study at one month in captivity. All of the fish examined from group 1 (N = 30 of 123) and group 2 (N = 15 of 40) were positive for the myxozoan by PCR (Table 1). Genomic DNA extracted from the stomach and intestine of healthy goby and used as negative control did not show any amplification using the PCR assay specific to the myxozoan. Freshly dead or moribund gobies from group 1 and fish sampled for histopathology from both groups were not included for estimating the parasite prevalence since these fish were specifically collected and used for spore extraction and identification, and histopathological evaluations.

**Table 1:** **Prevalence of myxozoan infections from yellowfin gobies as determined by PCR**

Number of fish	Total	Mortality	Histopathology	End of study	PCR	Myxozoan prevalence (%)
Group 1	151	18 (11.9%)	10 ^a^	123^*^	30/30^**^	100
Group 2	55	0	15 ^a^	40^*^	15/15^**^	100

^a^Sections were variable for myxozoan spores but difficult to confirm for vegetative stages by histopathology.

^*^Fish remaining at termination of the study at 1 month from which fish were randomly collected for PCR testing for presence of the myxozoan.

^**^Number of fish positive for the myxozoan per number of fish examined.

The spores obtained from the stomach and intestine of moribund gobies were almost spindle-shaped with mildly pointed anterior end. One sporoplasm was present in the spore body with a caudal appendage. Two almost equal polar capsules occupied most half of the spore cavity. The mean dimension of the spores (N = 10): spore body = 5.1 × 2.4 μm, tail = 9.9 μm, and polar capsule = 2.0 × 1.0 μm. Compared to other species of *Henneguya* found in goby species with body length ranging from 14.2 – 17.8 μm (Kageyama et al. 2009), the yellowfin goby myxozoan is significantly smaller.

**Figure 2:**
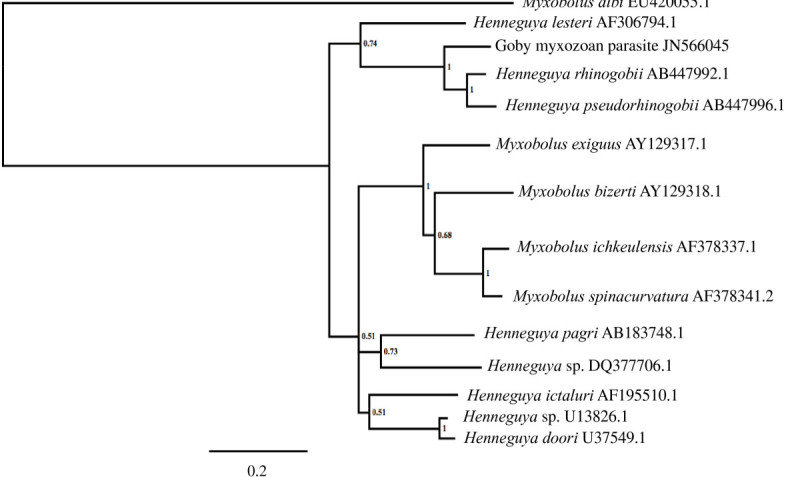
**Phylogenetic tree of the yellowfin goby myxozoan and closely related myxozoans using the 18S ribosomal DNA.** The tree was generated by MrBayes program ver. 3.1.2 based on the 18S rDNA long sequences (ca. 1.3 kb) covering Domain I through III. Posterior probabilities are listed at each node. The branch lengths, indicated by bars, are based on the number of inferred substitutions. *Myxobolus albi* was chosen as the out-group.

The 18 rDNA sequence of the myxozoan (1,987 bp) showed the highest similarity to *Henneguya rhinogobii* and *H. pseudorhinogobii* found in fresh water goby *Rhinogobius* sp. in Japan (Kageyama et al. 2009). The sequence data were deposited in GenBank (Accession No. JN566045). The goby myxozoan also showed the highest similarity with *H. pseudorhinogobii* and *H. rhinogobii* by pairwise comparison of the 18S rDNA Domain III (97.9%) and the long region covering Domain I through III (83–84%), which includes three variable regions (Table 2). The percentage similarity scores, especially for Domain III, further support that the goby parasite is closely related to the two myxozoans *H. pseudorhinogobii* and *H. rhinogobii* from Japanese gobies (Kageyama et al. 2009). However, the phylogenetic tree showed that the yellowfin goby myxozoan clearly branched separately as supported by high posterior probability value (1.00) suggesting a different species (Figure 2).

**Table 2:** **Pairwise comparison of the yellowfin goby myxozoan with other closely-related myxozoans shown in Table** 3

	Species	Accession no.	1	2	3	4	5	6	7	8	9	10	11	12	13	14
1	Goby myxozoan parasite	JN566045		84.6	83.7	74.4	73.2	75.2	74.5	71.1	71.5	72.4	69.9	72.3	71.2	58.3
2	*Henneguya rhinogobii*	AB447992.1	97.9		91.7	74.5	73.7	72.8	73.4	70.8	71.5	71.5	70.5	73.0	71.3	58.1
3	*H. pseudorhinogobii*	AB447996.1	97.9	99.2		74.0	73.6	73.0	72.8	71.3	72.1	70.8	71.0	72.4	72.2	57.9
4	*Henneguya ictaluri*	AF195510.1	92.0	92.2	92.0		73.7	76.6	79.5	74.2	74.6	77.2	74.7	75.4	74.5	59.7
5	*Henneguya lesteri*	AF306794.1	91.9	92.4	92.1	91.3		72.0	74.5	71.9	72.5	72.3	70.6	72.2	71.4	57.3
6	*Henneguya pagri*	AB183748.1	91.9	91.9	92.3	94.6	90.5		76.8	75.8	75.6	74.5	73.6	76.5	74.6	58.1
7	*Henneguya* sp.	U13826.1	91.4	91.2	91.0	93.6	89.6	92.7		75.0	74.4	92.7	73.9	76.1	74.1	58.6
8	*Myxobolus ichkeulensis*	AF378337.1	90.8	90.4	90.4	93.0	91.2	93.2	91.6		91.9	73.0	80.3	73.0	81.1	58.7
9	*Myxobolus spinacurvatura*	AF378341.2	90.2	90.7	90.9	93.1	91.5	93.2	91.2	97.9		72.7	80.7	72.7	80.9	58.7
10	*Henneguya doori*	U37549.1	90.1	90.0	89.8	92.3	88.4	91.5	96.9	90.3	90.0		72.5	74.6	72.3	57.3
11	*Myxobolus bizerti*	AY129318.1	90.0	90.0	90.2	92.2	90.1	92.1	91.0	97.0	97.0	89.8		72.0	79.4	58.2
12	*Henneguya* sp.	DQ377706.1	88.9	89.2	89.1	90.9	88.2	91.8	88.6	89.2	89.0	87.4	87.6		72.9	57.8
13	*Myxobolus exiguus*	AY129317.1	88.5	88.3	88.7	90.9	89.5	91.1	88.8	94.1	94.4	87.8	94.2	86.7		58.2
14	*Myxobolus albi*	EU420055.1	74.9	74.7	74.9	76.0	73.7	75.0	74.5	75.5	75.4	73.8	75.3	73.1	74.3	

Note: The scores are similarity in percentage. Bottom left: short conserved regions (Domain III, ca. 540 bp). Top right: long fragments (Domain I through III, ca 1.3 kb) of the 18S rDNA sequences.

## Discussion

The goby myxozoan is consistent with the description of the family Myxobolidae and the genus *Henneguya* based on morphological criteria of the spore stages (Lom and Arthur 1989Lom and Dykova 1992,2006). The taxonomic identification of the goby myxozoan was confirmed by molecular analyses showing close similarity to *H. rhinogobii* and *H. pseudorhinogobii* previously reported from the Japanese fresh water goby (Kageyama et al. 2009). Confirmatory identification of the goby myxozoan in the genus *Henneguya* is based on the pairwise comparison of the 18S rDNA conserved region (Domain III), which is 97.9% similar with *H. rhinogobii* and *H. pseudorhinogobii* and from phylogenetic analysis by forming a clade with both species of *Henneguya* from the Japanese goby. While high similarity values were observed among the domain III of the Japanese goby and the yellowfin goby myxozoans, the sequence similarity of the long region (Domain I through III, 83–84%) and the phylogenetic tree suggests the yellowfin goby myxozoan is a different species. The morphologic features (small spore size) further indicate the yellowfin goby myxozoan is different from the freshwater goby myxozoan (Kageyama et al. 2009). An in-depth morphological description of the spores and vegetative stages is currently lacking to support the proposal to a new species of *Henneguya*. The goby myxozoan will therefore be referred to as *Henneguya* sp. until additional morphological data are available to support its classification to a new species.

Because myxozoan spores were present in dead, moribund, and in some apparently healthy gobies that were examined, infections were contracted prior to their collection from the field. In this context, gobies may provide reservoirs of infection at Suisun Marsh although the exact mechanism and the pathogenicity of the goby myxozoan have yet to be determined. Considered the second largest genus within Myxosporea, *Henneguya* is one of the most important groups of pathogens affecting both freshwater and marine fishes (Lom and Dykova 1992). Certain myxozoan species are known agents of serious diseases in fish (Kent et al. 2001). *Henneguya* infections occur mainly on the gills rendering respiratory failure and mortality due to asphyxia (Lom and Dykova 1992El-Mansy 2002). The *Henneguya* sp. was not observed in the gill tissues of gobies examined in this study. The only *Henneguya* species found in intestinal tissues of goby is *H. rhinogobii* found in the goby *Rhinogobius giurinus* from China (Kageyama et al. 2009). While the myxozoan was prevalent from gobies in both groups, mortalities (N = 18) only occurred in the first group with smaller size fish. Histopathological changes associated with the parasite were not observed among moribund gobies in the first group. Exposure experiments are warranted to determine if the mortalities are directly attributed to the myxozoan and if certain life stages of the gobies are more susceptible to infections. For these reasons, the significance of the myxozoan as a potential pathogen is currently unknown.

In their native habitats, gobies feed on small fish, benthic crustaceans, and worms (Kikuchi and Yamashita 1992Hironouchi and Sano 2000Workman and Merz 2007). Fish and oligochaetes are the fundamental hosts in the myxozoan life cycle (Kent et al. 2001). When these hosts die, they liberate spores into the water column that are infectious to the other host (Hedrick et al. 1998Kent et al. 2001). At present, the mode of transmission and geographic source of the parasite as contracted by the goby in Suisun Marsh have yet to be determined. Horizontal transmission has been shown only in the marine myxozoan *Enteromyxum leei* (Diamant 1997). Except for *E. leei*, parasite transmission precluding an obligate alternate host has not been demonstrated for other myxozoans.

Whether this pathogen was introduced by the goby from its native origin in Japan or whether the goby contracted it from its current environment (e.g. water column, sediment, infected preys such as worms being a part of their natural diet) is unknown. However, based on the presence of similar parasites from gobies in Japan, it seems likely that the parasite invaded along with its host. Furthermore, the similarity score in the 18S rDNA Domain III is very high (97.9%) between the gobiid myxozoans from the two distant locations.

Of interest in the myxozoan is their relevance in the abundance of the yellowfin goby being an introduced species in the SFE. It is important to note that monitoring for pathogens and diseases has not been a focus of the various fish surveys in the Sacramento San Joaquin Delta. Interestingly, monitoring programs in Suisun Marsh conducted from 1979 to 2007 demonstrates the peak of goby abundance from 1992 to 2001 but significantly declined from 2002 (Meng et al. 1994Matern et al. 2002;O’Rear and Moyle , O’Rear and Moyle O’Rear and Moyle 2010). Invasion ecology suggests that disease can either reduce (if it affects the invader) or increase (if initiated by a relatively immune invader) the impact of introduced species (Simberloff and Gibbons 2004). While many pathogens are known as disease agents in captive fish populations, the effects of diseases on wild populations are notoriously difficult to assess due to the complex interactions of many variables in the aquatic environment (Hedrick 1998). In a stressed ecosystem as the SFE, invasive species harboring exotic pathogens may affect the abundance of the host population by carrying pathogens deemed more pathogenic to naïve hosts (Lafferty et al. 2004Riley et al. 2008). Effects on hosts may be broad if the disease can infect new species that have no means of avoiding or reducing infection (Lafferty et al. 2005). Empirical evidence on the potential impacts of invasive species harboring pathogens and diseases in natural systems is unknown. Environmental factors however, may help create unique conditions for alien organisms to dominate and out-compete native species (Brook 2008Bradley et al. 2010).

## Conclusion

This study documents the occurrence of a potentially new species of myxozoan in the yellowfin goby and underscores the detection of a parasitic infection in an introduced fish in the San Francisco Estuary. Although the significance of this parasite as a potential pathogen is unknown, the myxozoan may alter the performance and survival of yellowfin gobies by blocking the linings of the intestine and stomach. The PCR assay that we developed will provide a specific and rapid diagnostic tool to identify carriers of the myxozoan. PCR screening of species that may harbor the parasite and histopathological assessment on the severity of infections will provide a better understanding of the parasite impact on the long-term health of gobies and the potential transmission of infections to other susceptible fish species. While the prevalence of the myxozoan as reported here is restricted to a comparatively small collection site, the reemergence, identification, and ecological relevance of the parasite on goby populations in the San Francisco Estuary may be investigated in the future using the specific diagnostic tool developed in this study.

## Methods

**Figure 3:**
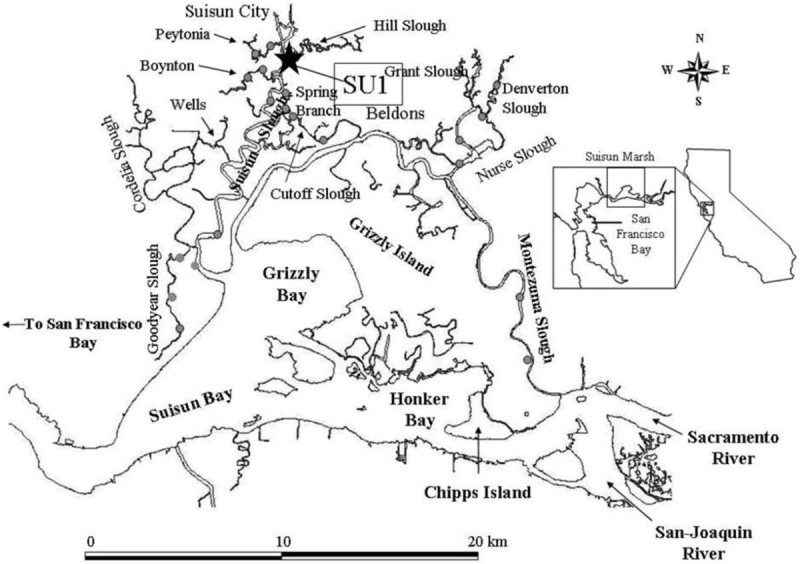
**Map of Suisun Marsh.** The sampling location of yellowfin goby in June and July 2005 in northern Suisun slough is indicated as SU1.

Two groups of juvenile gobies were collected in June and July 2005 in the northern part of Suisun slough at Suisun Marsh (Figure 3) as part of a monitoring survey of juvenile fishes. The gobies were transported live to the Center for Aquatic Biology and Aquaculture (CABA) at UC Davis in aerated tanks containing ambient water (22 ± 1°C) from the marsh. Gobies in the first group (N = 151, average size: 1.5 gm, 50 mm) were collected in June 2005 at Suisun slough by beach seining. The second group (N = 55, average size: 10.0 gm, 100 mm) were sampled in the same location in July 2005 using otter trawl and beach seine. The two groups were held separately in aerated 130-L tanks receiving (22–23°C) non-recirculating well water at CABA, UC Davis for one month.

After collection from the estuary, moribund gobies (N = 5) were observed from the first group and were therefore collected to determine if an agent is likely associated with the morbidity. Various organs such as the gill, spleen, and kidney were initially examined from these fish by wet mounts and did not reveal any parasitic stages. However, the stomach and intestinal tissues showed the presence of spores by light microscopy and were therefore extracted and purified by Percoll method (Hamilton and Canning 1988). The purified spores were used for analysis of morphologic features (Lom and Arthur 1989Lom and Dykova 2006) and for DNA extraction for identification by molecular approaches and to develop a specific PCR. Moribund gobies were euthanized with ethyl-*p*-aminobenzoate (500 μg/L, Benzocaine, Sigma) prior to dissection for observation of spores from stomach and intestinal tissues. Spores were measured for dimensions and features following the guidelines for myxozoan species (Lom and Arthur 1989). Gobies were also collected (group 1: N = 10, group 2: N = 15) and processed for histopathology (Humason 1979) and stained with hematoxylin and eosin. These samples were also PCR tested using the primers and conditions as described below. Gobies were euthanized with benzocaine as above prior to processing for PCR assays and fixing in buffered formalin for histopathology.

Genomic DNA (gDNA) was extracted from spores using a QIAamp DNA Mini kit (Qiagen). The 18S ribosomal gene (rDNA) region was PCR-amplified using a universal primer set (18e–18 g’) targeting ca. 1,900 bp fragment (Hillis and Dixon 1991Andree et al. 1999). The PCR products were purified (QIAEX II, Qiagen), and ligated into pGEM-T Easy vector (Promega) for transformation (Invitrogen). The plasmids containing the PCR amplified DNA fragment were sequenced using an ABI 377 automated DNA sequencer (Applied Biosciences).

The entire 18S rDNA sequence of the myxozoan obtained in this study (1,987 bp) was used for similarity search using the BLASTN program (http://www.ncbi.nlm.nih.gov/). The conserved (Domain III, ca. 540 bp) and long regions (Domain I through III including variable regions, 1.3 kb) of the sequence were selected for further analyses. The multiple sequence alignments were generated for each of the region for the goby myxozoan and closely-related myxozoans listed in Table 3 using MUSCLE ver. 3.8.31 (Edgar 2004). This was followed by pairwise comparison using Geneious ver. 6.1 Drummond et al. 2011). The phylogenetic tree was generated by MrBayes program (ver. 3.1.2) for Domain I − III (1.3 kb) using Markov chain Monte Carlo method with the following settings: Ngen = 10000000, Nchain = 4, Temp = 0.5, Stopval = 0.01, Samplefreq = 50, Printfreq = 1000 (Ronquist and Huelsenbeck 2003). The general time-reversible (GTR) model with gamma-distributed rates was used for the analysis as chosen by jModelTest ver. 2.1.4 (Darriba et al. 2012). *Myxobolus albi*, a closely related myxozoan that does not belong to *Henneguya* spp., was used as an out-group for the phylogenetic tree analysis. The phylogenetic trees were depicted by FigTree ver 1.3.1 (http://tree.bio.ed.ac.uk/software/figtree/).

**Table 3:** **Myxozoan parasites used for pairwise comparison with the yellowfin goby myxozoan**

Species	Host	Location	Accession no.	Domain III (540 bp)	Domain I-III (1.3 kb)	Reference
Goby myxozoan parasite	Yellowfin goby	California, USA	JN566045	978-1,520	413-1,750	This study
*Henneguya doori*	Yellow perch	Halifax, Canada	U37549.1	894-1,440	362-1,661	Siddall et al. 1995
*Henneguya ictaluri*	Channel catfish	Mississippi, USA	AF195510.1	947-1,488	412-1,717	Hanson et al. 2001
*Henneguya lesteri*	Unknown	Unknown	AF306794.1	984-1,527	418-1,758	Unpublished data
*Henneguya pagri*	Red sea bream	Japan	AB183748.1	956-1,499	417-1,732	Yokoyama et al. 2005
*Henneguya pseudorhinogobii*	Freshwater goby	Nagara River, Japan	AB447996.1	895-1,437	333-1,663	Kageyama et al. 2009
*Henneguya rhinogobii*	Freshwater goby	Nagara River, Japan	AB447992.1	891-1,433	331-1,660	Kageyama et al. 2009
*Henneguya* sp.	Palometa	Caribbean Sea, Mexico	DQ377706.1	979-1,527	427-1,751	Fiala 2006
*Henneguya* sp.	Mottled sculpin	Unknown	U13826.1	929-1,473	394-1,707	Smothers et al. 1994
*Myxobolus albi*	Common goby	Forth Estuary, Scotland	EU420055.1	493-1,033	1-1,189	Picon-Camacho et al. 2009
*Myxobolus bizerti*	Mullet	Ichkeul Lake, Tunisia	AY129318.1	844-1,386	367-1,591	Bahri et al. 2003
*Myxobolus exiguus*	Mullet	Ichkeul Lake, Tunisia	AY129317.1	841-1,382	305-1,587	Bahri et al. 2003
*Myxobolus ichkeulensis*	Mullet	Ichkeul Lake, Tunisia	AF378337.1	839-1,382	300-1,590	Bahri et al. 2003
*Myxobolus spinacurvatura*	Mullet	Ichkeul Lake, Tunisia	AF378341.2	787-1,327	251-1,533	Bahri et al. 2003

Note: The location of the conserved region of the 18S rDNA (Domain III) of the myxozoans (Picon-Camacho et al. 2009) is shown. The short conserved regions (Domain III, ca. 540 bp) and the long fragments (Domain I through III, ca 1.3 kb) of the 18S rDNA sequences were both used for pairwise comparison while the phylogenetic analysis, used only the long fragments.

The goby myxozoan parasite was detected by developing a specific PCR assay targeting a unique region of the 18S rDNA. A primer pair, Goby Myx 2 F (5’-ATG CTT CCG GGT ACT GTA GG-3’) and Goby Myx 2R (5’-CAC GCT CGT GAG AAC GAT TC-3’) generated a 150 bp PCR product. The PCR cocktail (Invitrogen) for a 50 μl reaction contained 200 μM of dNTPs, 1.5 mM of MgCl_2_, 40 pmol of each primer, 1 unit Platinum® Taq DNA polymerase, and 10x buffer at 1/10 the volume. The PCR cycle profile was performed consisting of an initial denaturation step of 95°C for 5 min, 40 cycles of 95°C for 30 s, 55°C for 1 min, 72°C for 30 s, and a final extension step at 72°C for 5 min. The specificity of the PCR test was verified by demonstrating the inability to amplify 18S rDNA fragment from other closely related myxozoans including *Myxobolus* sp. and *M. cerebralis*.

The prevalence of the myxozoan among the collected gobies was assessed at termination of the study after one month in captivity by randomly collecting gobies from the first group (30 from a total of 123 remaining fish) and from the second group (15 from a total of 40 remaining fish). Gobies that were moribund or dead and fish used for histopathological analysis were not included for estimating the myxozoan prevalence in the two groups. Apparently healthy gobies were also processed as negative controls. The sampled gobies from both groups and the controls were euthanized with 500 μg/L benzocaine and were processed for diagnostic PCR testing using the primers and conditions described above. Genomic DNA was extracted from pooled stomach and intestinal tissues of each fish and subjected to the reaction following the optimized condition. Amplified DNA fragments were randomly chosen and processed for direct sequencing at Davis Sequencing Service to confirm the parasite DNA sequence.

## Authors’ information

DVB, TK, SJT are members of the Aquatic Health Program at UC Davis, School of Veterinary Medicine: http://www.vetmed.ucdavis.edu/aquatic_health/index.cfm.

## Authors’ original submitted files for images

Below are the links to the authors’ original submitted files for images. Authors’ original file for figure 1 Authors’ original file for figure 2 Authors’ original file for figure 3
