# Supplementary figures and images for: Henneguya sp. in yellowfin goby Acanthogobius flavimanus from the San Francisco Estuary
Source: Springerplus. 2013 Aug 29;2(1):420. doi: 10.1186/2193-1801-2-420 (PMC3765604; doi:10.1186/2193-1801-2-420)

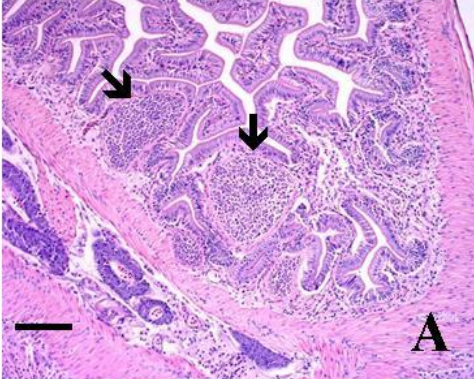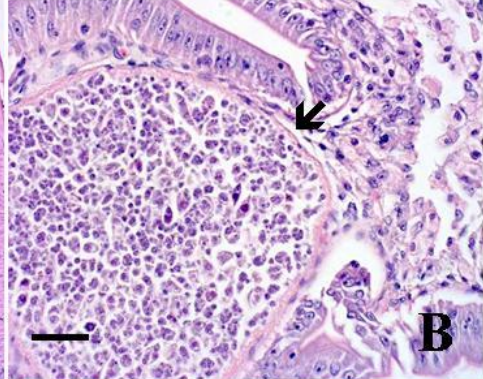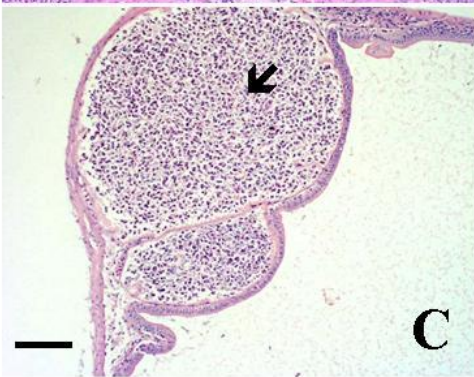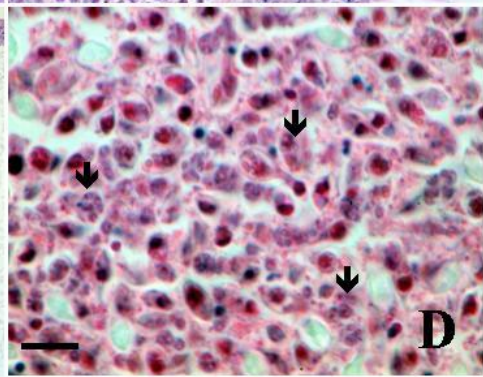

Supplement: Supplementary file 1 — Authors’ original file for figure 1 [file 40064_2013_484_MOESM1_ESM.pdf]

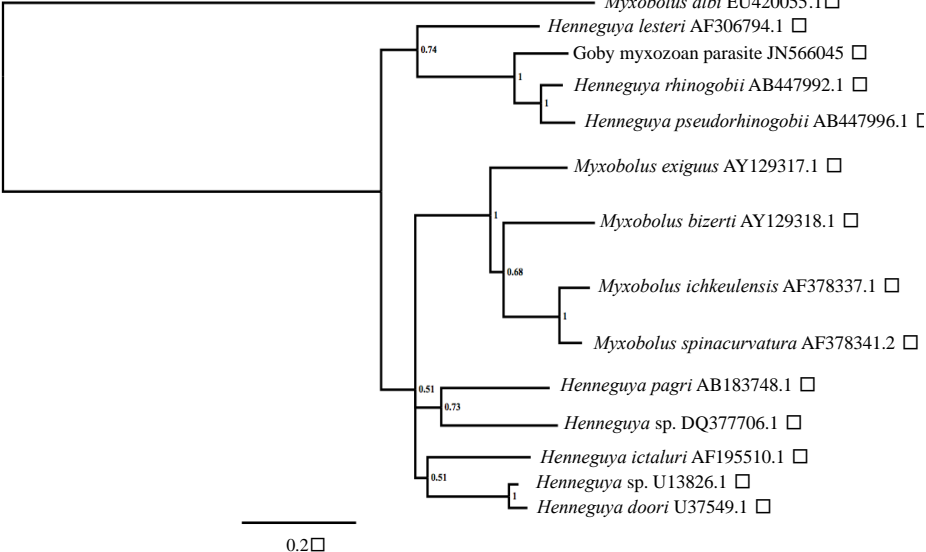

Supplement: Supplementary file 2 — Authors’ original file for figure 2 [file 40064_2013_484_MOESM2_ESM.pdf]

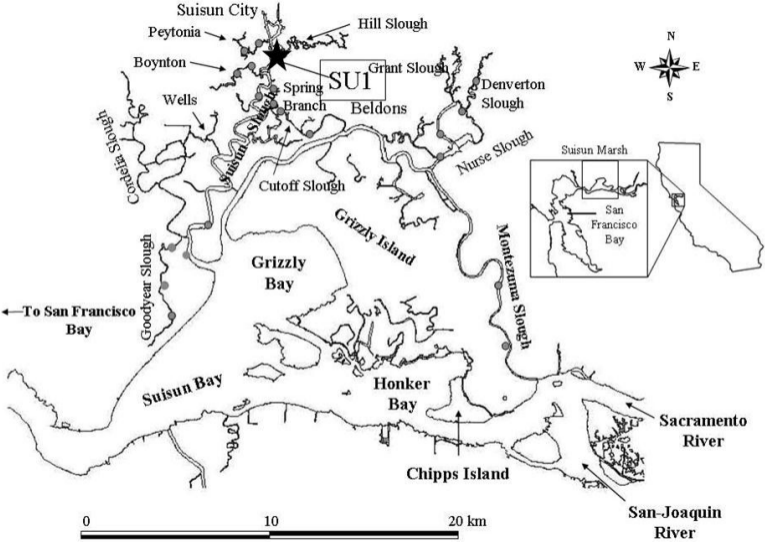

Supplement: Supplementary file 3 — Authors’ original file for figure 3 [file 40064_2013_484_MOESM3_ESM.pdf]
